# Preclinical Evaluation of Triptophenolide-Induced Apoptosis in Hepatoblastoma (HepG2) and Hepatocellular Carcinoma (HuH7) Cell Lines

**DOI:** 10.3390/ijms27073251

**Published:** 2026-04-03

**Authors:** Zufa Sabeel, Ruolan Chen, Yan Liu, Xiaoyang Chen, Wenjing Zhang, Shangyang Pan, Lu Ying, Changyuan Yu, Zhao Yang

**Affiliations:** College of Life Science and Technology, State Key Laboratory of Green Biomanufacturing, Innovation Center of Molecular Diagnostics, Beijing University of Chemical Technology, Beijing 100029, China

**Keywords:** liver cancer, Triptophenolide, antitumor effect, proliferation, apoptosis, xenograft model

## Abstract

Liver cancer is one of the most prevalent and lethal cancers worldwide, characterized by poor prognosis and limited treatment options. Triptophenolide (TRI), a diterpenoid compound, has shown anti-proliferative activity in breast and pancreatic cancers, but its role in liver cancer remains largely unexplored. In this study, TRI significantly inhibited the proliferation of HepG2 (hepatoblastoma) and HuH7 (hepatocellular carcinoma) cells in a dose-dependent manner, with IC_50_ values decreasing from 279.9 to 229.4 µg/mL (24–48 h) in HepG2 and from 441.1 to 282.6 µg/mL in HuH7. Colony formation assays confirmed the suppression of HCC cell growth. TRI also promoted apoptosis, increasing apoptotic rates to 68.99% in HepG2 and 43.34% in HuH7 at 400 µg/mL (48 h). Cell cycle analysis revealed S-phase arrest, with TRI raising the S-phase population to 42.02% and 45.38%, respectively. Mechanistically, TRI upregulated pro-apoptotic genes (*TP53*, *CASP3*/*9*/*10*, *BAX*, *BAK1*, *BID*, *BIM*) and proteins, activating the mitochondrial apoptotic pathway. In vivo, TRI (10 mg/kg) markedly reduced tumor volumes in HepG2 and HuH7 xenografts compared with controls, without obvious systemic toxicity. These findings suggest that TRI exerts anti-proliferative, pro-apoptotic, and cell cycle regulatory effects in HCC. However, further preclinical studies are warranted to elucidate its mechanisms and evaluate its safety profile.

## 1. Introduction

Worldwide, primary liver cancer accounts for the third highest number of cancer deaths [1]. It presents a major health challenge across all age groups, from the most frequent pediatric form, hepatoblastoma, whose incidence is rising [2], to the most common adult malignancy, hepatocellular carcinoma (HCC) [3]. Despite regional variations in incidence and mortality, the global burden of liver cancer continues to increase, with projections estimating a substantial rise in both incidence and death rates by 2040 [4,5]. Chronic liver diseases, including hepatitis B and C virus infection, alcohol-related liver disease, metabolic dysfunction-associated steatotic liver disease (MASLD), and autoimmune or cholestatic liver disorders, represent the major etiological risk factors for primary liver cancer development [6]. Due to its aggressive nature and frequent diagnosis at advanced stages, liver cancer remains associated with poor clinical outcomes and significant societal and healthcare burdens [7,8].

Despite advances in cancer treatments, liver cancer outcomes remain poor, particularly for those diagnosed at later stages. Standard therapies like surgical resection, liver transplants, and chemotherapy have limited success and can cause severe side effects [9]. Transarterial chemo-embolization (TACE), and hepatic arterial infusion chemotherapy (HAIC) are commonly used for advanced or inoperable liver cancer but primarily address localized liver tumors [10]. Systemic chemotherapeutics, including doxorubicin or tamoxifen have shown limited survival benefits [11,12], and alternative strategies are needed for cisplatin-resistant liver cancer [10]. These challenges underscore the need to develop more effective and less toxic anticancer agents [13].

Natural compounds and their derivatives have promising avenues for anticancer therapy due to their diverse biological activities and generally favorable safety profiles [14,15,16,17]. Among these, TRI, a diterpenoid isolated from *Tripterygium wilfordii Hook. f.* (TW), exhibits anticancer, anti-androgen, and anti-inflammatory properties [18]. Bioactive components of TW have demonstrated anticancer activity across a range of tumors, including liver, lung, oral, breast, lymphoma, and leukemia [19,20,21,22,23]. Notably, TRI was identified as a key anticancer component in a TW and *Scutellaria barbata D. Don* combination that potently suppressed HepG2 cells [22]. Importantly, TRI did not exhibit hepatotoxicity in zebrafish models, suggesting a relatively safer profile among TW constituents [24].

Previous studies have indicated that TRI exhibits anti-inflammatory effects [25] and is capable of regulating T lymphocyte–mediated immune responses [26]. While several bioactive constituents of TW, including triptolide and tripterine, have been extensively studied for their pharmacological activities [27,28,29], TRI has received comparatively less attention [26]. Notably, although triptolide shows strong anticancer activity, its therapeutic use is limited by significant multi-organ toxicity, including hepatotoxicity [24,30]. Recent investigations continue to elucidate the mechanisms of triptolide-induced liver injury, demonstrating, for example, that it can elevate liver enzymes (AST, ALT), induce liver bleeding, and activate inflammatory pathways [31]. Interestingly, the same study reported that another TW component, TRI, was safe to the liver and intestine when administered alone, but its combination with triptolide exacerbated the injuries caused by triptolide [31]. In contrast, TRI has been reported to exhibit moderate cytotoxic effects along with a potentially improved safety profile [24]. These characteristics suggest that TRI may warrant further investigation as a therapeutic candidate. However, its potential anticancer activity and the molecular mechanisms involved in liver cancer have not yet been clearly defined.

To date, no studies have systematically explored TRI-induced programmed cell death pathways in liver cancer. Therefore, this study investigates how TRI, as a single agent, induces apoptosis and affects cell cycle progression in liver cancer cells, aiming to elucidate its underlying molecular mechanisms. By providing these mechanistic insights, this work offers preclinical evidence supporting TRI as a candidate for further investigation in liver cancer research.

## 2. Results

### 2.1. TRI Suppressed HepG2 and HuH7 Cell Proliferation

**Figure 1:**
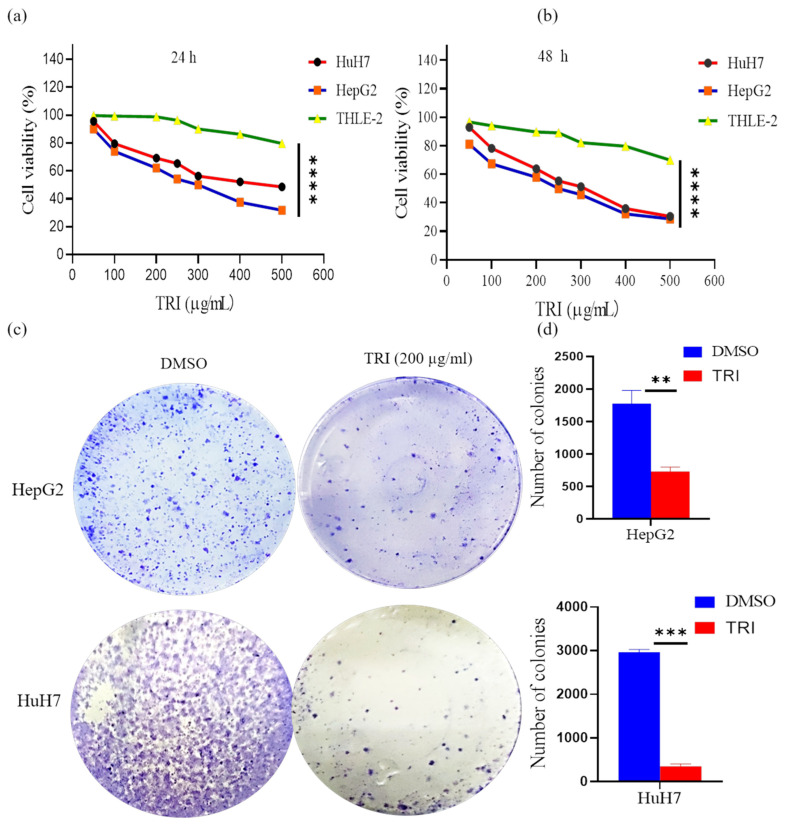
TRI suppressed proliferation and clonogenic potential in liver cancer. (**a**) Cell viability of HepG2 and HuH7 cells treated with increasing doses of TRI for 24 h, as measured by CCK-8 assay. (**b**) Cell viability after 48 h of TRI treatment, demonstrating enhanced growth inhibition. (**c**) Representative images of colony formation assays for HepG2 and HuH7 cells following treatment with vehicle control or the indicated concentrations of TRI for 48 h. (**d**) Quantitative analysis of colony formation assays confirmed a significant suppression of clonogenic survival in both cell lines. Data are presented as mean ± SD (*n* = 3 independent experiments). ** *p* < 0.001, *** *p* < 0.001, **** *p* < 0.0001, versus the control group.

Cell proliferation is a key indicator of cellular growth activity. The antiproliferative effects of TRI were quantitatively assessed using CCK-8 assays in liver cancer cell lines (HepG2, HuH7) and normal hepatocytes (THLE-2 cells). As shown in Figure 1a,b, TRI exhibited potent, dose-dependent antiproliferative effects in liver cancer cell lines while demonstrating minimal cytotoxicity toward normal hepatocytes. Notably, a consistent reduction in cell viability was observed across both HepG2 and HuH7 cells, indicating that the inhibitory effect of TRI is reproducible across distinct liver cancer models. This consistent response across biologically distinct liver cancer cell lines suggests that TRI may exert broad anti-tumor activity.

Quantitative analysis revealed that 24 h treatment (50–500 μg/mL) reduced HepG2 viability from 90.12 ± 2.34% to 31.87 ± 3.21% (*p* < 0.001) and HuH7 viability from 95.51 ± 1.89% to 48.52 ± 2.76% (*p* < 0.01), whereas normal THLE-2 cells maintained 79.69 ± 4.53% viability (Figure 1a). Prolonged exposure to 48 h enhanced these effects, with HepG2 viability declining to 28.65 ± 2.98% and HuH7 to 30.46 ± 3.45%, while THLE-2 cells retained 69.88 ± 3.89% viability (*p* < 0.05 versus liver cancer cells; Figure 1b). IC_50_ calculations demonstrated dose-dependent potency, decreasing from 279.9 μg/mL (24 h) to 229.4 μg/mL (48 h) in HepG2 cells and from 441.1 μg/mL to 282.6 μg/mL in HuH7 cells. Additionally, to assess the long-term impact on cell proliferation, a clonogenic survival assay was conducted. As shown in Figure 1c,d, TRI treatment markedly suppressed colony formation in HepG2 and HuH7 cells relative to the DMSO control group. These results support the conclusion that TRI can inhibit the proliferative capacity of liver cancer cells.

### 2.2. TRI Induced Apoptosis in Liver Cancer Cells

**Figure 2:**
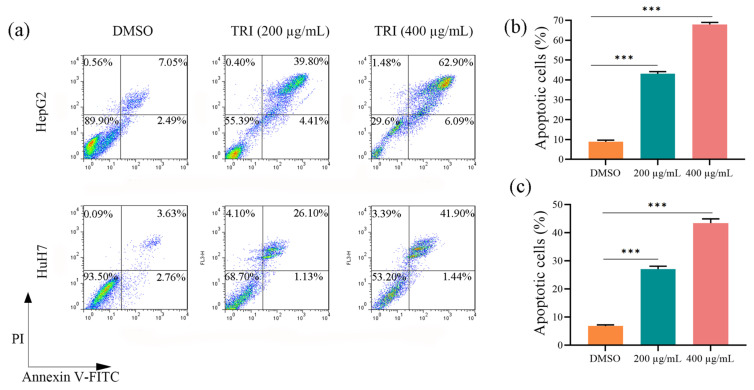
TRI induces apoptosis in liver cancer cells. (**a**) Flow cytometry analysis of apoptotic cells using Annexin V-FITC/PI double staining. (**b**) Quantitative apoptosis rates in HepG2 cells. (**c**) Quantitative apoptosis rates in HuH7 cells. Data represent mean ± SD of three independent experiments. *** *p* < 0.001 versus untreated control (one-way ANOVA with Tukey’s post hoc test).

Flow cytometric analysis using Annexin V-FITC/PI staining demonstrated significant apoptosis induction by TRI in liver cancer cell lines (Figure 2a–c). Treatment with 200 μg/mL for 48 h elevated apoptotic rates from 9.54 ± 1.23% to 44.21 ± 3.45% in HepG2 cells (4.63-fold, *p* < 0.001; Figure 2b) and from 6.39 ± 0.98% to 27.23 ± 2.76% in HuH7 cells (4.26-fold, *p* < 0.01; Figure 2c). At 400 μg/mL, apoptosis increased to 68.99 ± 4.12% (7.23-fold, *p* < 0.001) and 43.34 ± 3.89% (6.78-fold, *p* < 0.001) respectively, showing dose-dependence (r^2^ = 0.94–0.97). The observed quantitative differences in apoptotic rates may reflect intrinsic variability in the molecular and genetic backgrounds of these two liver cancer cell lines. HepG2 cells, derived from a hepatoblastoma [32], and HuH7 cells, derived from a well-differentiated hepatocellular carcinoma [33], exhibit distinct gene expression profiles. Notably, the similar response patterns between cell lines (*p* > 0.05 at equivalent doses) suggest that TRI activates conserved apoptotic mechanisms across liver cancer subtypes.

### 2.3. TRI Arrested Cell Cycle at S Phase in Liver Cancer Cells

**Figure 3:**
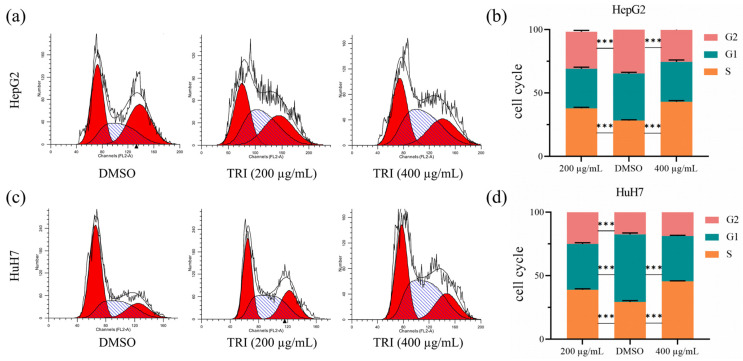
TRI induces cell cycle arrest in liver cancer cells. (**a**) Cell cycle distribution of TRI-treated HepG2 cells by flow cytometry. (**b**) Quantitative analysis of HepG2 cell cycle phases. (**c**) Cell cycle distribution of TRI-treated HuH7 cells by flow cytometry. (**d**) Quantitative analysis of HuH7 cell cycle phases. In the flow cytometry histograms, the left red peak represents cells in the G0/G1 phase, the central white shaded region represents cells in the S phase, and the right red peak represents cells in the G2/M phase. Data represent mean ± SD of three independent experiments. *** *p* < 0.001 vs. untreated control (one-way ANOVA with Tukey’s post hoc test).

Cell cycle analysis via propidium iodide (PI) staining indicated that TRI treatment resulted in a significant accumulation of cells in S phase (Figure 3). In HepG2 cells, exposure to 200 μg/mL TRI increased the S-phase population from 27.68 ± 2.12% to 38.69 ± 3.01% (*p* < 0.01). Treatment with 400 μg/mL further increased the proportion of cells in S phase to 42.02 ± 3.45% (*p* < 0.001) (Figure 3a,b). A comparable effect was observed in HuH7 cells, with the S-phase fraction rising from 29.81 ± 2.34% to 39.59 ± 2.89% (200 μg/mL, *p* < 0.01) and to 45.38 ± 3.12% (400 μg/mL, *p* < 0.001) (Figure 3c,d). This dose-dependent accumulation of cells in S phase, representing an absolute increase of 11.01–15.57%, is consistent with an impairment of DNA synthesis and cell cycle progression. As therapies that induce S-phase arrest are known to preferentially target proliferating cells, this mechanism may be a key contributor to the anti-proliferative efficacy of TRI observed in this study.

### 2.4. TRI Induced Apoptosis Through Transcriptional and Translational Activation of the Mitochondrial Pathway

**Figure 4:**
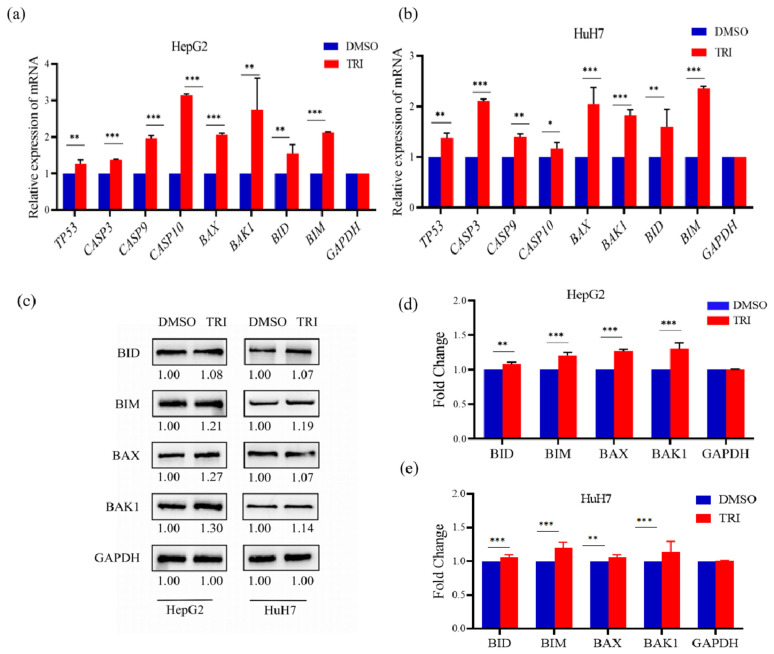
TRI induces mitochondrial apoptosis in liver cancer cells through transcriptional and translational regulation. (**a**) qRT-PCR analysis of apoptosis-related genes in HepG2 cells and (**b**) HuH7 cells after TRI treatment. (**c**) Representative Western blots and quantitative analysis of key apoptotic proteins in (**d**) HepG2 and (**e**) HuH7 cells following TRI treatment. Data shown as mean ± SEM from three biological replicates * *p* < 0.05, ** *p* < 0.01, *** *p* < 0.001 vs. vehicle control.

TRI induced apoptosis in liver cancer cells through coordinated transcriptional and translational activation of mitochondrial apoptotic pathways. qRT-PCR analysis revealed that TRI treatment significantly upregulated *TP53* expression by 1.23-fold in HepG2 cells (*p* < 0.05) and 1.45-fold in HuH7 cells (*p* < 0.01), consistent with its role in initiating intrinsic apoptosis (Figure 4a,b). Caspase cascade components exhibited cell-type-specific responses, with *CASP3* increasing by 1.36-fold in HepG2 and 2.07-fold in HuH7, *CASP9* increasing by 2.00-fold in HepG2 versus 1.46-fold in HuH7, and *CASP10* showing pronounced induction in HepG2 (3.10-fold) compared to HuH7 (1.12-fold). Bcl-2 family proteins involved in mitochondrial outer membrane permeabilization (MOMP) were also transcriptionally upregulated, including BAX (2.09-fold HepG2; 1.76-fold HuH7), BAK1 (3.72-fold HepG2; 1.94-fold HuH7), and the BH3-only proteins BID (1.39–1.52-fold) and BIM (2.10–2.30-fold) (Figure 4a,b).

Western blot analysis of key apoptosis regulators was performed to examine protein expression changes (Figure 4c–e). TRI treatment resulted in significant upregulation of pro-apoptotic proteins in both cell lines, though with distinct expression patterns. In HepG2 cells, BAK1 showed the most substantial increase (1.30 ± 0.08-fold, *p* < 0.01), followed by BAX (1.27 ± 0.05-fold), BIM (1.18 ± 0.03-fold), and BID (1.08 ± 0.02-fold) (all *p* < 0.05). HuH7 cells exhibited a different response, with BIM demonstrating the highest protein induction (1.19 ± 0.04-fold, *p* < 0.01) alongside more modest increases in BAK1 (1.14 ± 0.03-fold), BAX (1.12 ± 0.02-fold), and BID (1.07 ± 0.01-fold). These protein expression patterns corresponded with transcriptional changes observed in qRT-PCR analyses.

Collectively, these findings indicate that TRI promotes apoptosis through TP53-dependent signaling, enhanced mitochondrial pore formation, and subsequent caspase activation, with HepG2 cells showing greater sensitivity particularly in CASP10 induction (*p* < 0.001 versus HuH7). TRI modulated key apoptotic regulators at both transcriptional and translational levels, with observed cell line-specific variations in pathway activation (Figure 4a–e).

### 2.5. Effects of TRI Treatment in Liver Cancer Xenograft Models

**Figure 5:**
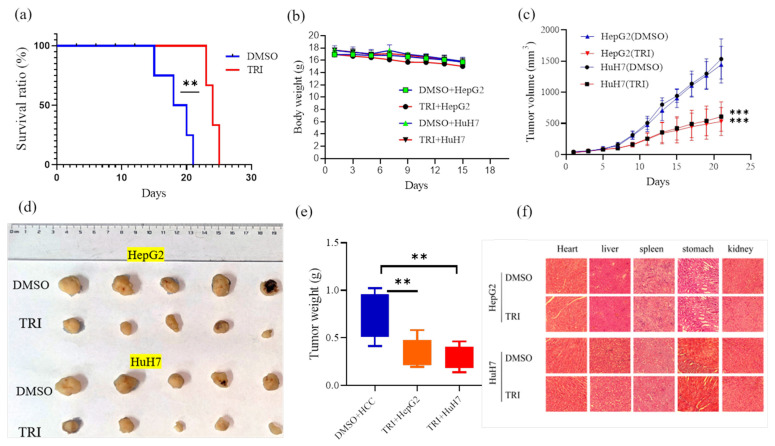
In vivo evaluation of TRI’s anti-tumor efficacy in HepG2 and HuH7 xenograft models. (**a**) TRI significantly improved survival. (**b**) No significant weight loss or toxicity was observed. (**c**) TRI strongly inhibited tumor growth. (**d**) Representative images of excised tumors. (**e**) TRI reduced tumor weight. (**f**) H&E staining of major organs showed no drug-related damage. Data are mean ± SD; statistical analysis by Student’s *t*-test or log-rank test. ** *p* < 0.01, *** *p* < 0.001 vs. vehicle control.

To evaluate the anti-tumor efficacy of TRI against liver cancer in vivo, we utilized xenograft models established in immunocompromised mice. TRI was administered at a dose of 10 mg/kg every other day for 21 days. TRI treatment significantly improved survival outcomes: all control animals succumbed to their tumor burden between days 14 and 20, while TRI-treated animals survived until the study endpoint on day 24 (*p* < 0.01; Figure 5a). No significant differences in body weight were observed between treated and control groups (*p* > 0.05; Figure 5b).

Treatment with TRI significantly inhibited the growth of both HepG2 and HuH7-derived tumors compared to vehicle controls (Figure 5c). At the endpoint, the final tumor volume in the TRI-treated group was reduced to 530.37 ± 45.21 mm^3^, compared to 1442.44 ± 132.67 mm^3^ in the control group for HepG2 xenografts (*p* < 0.001). A similar potent inhibition was observed in HuH7 xenografts, with volumes of 533.71 ± 68.34 mm^3^ versus 1493.94 ± 118.92 mm^3^ in controls (*p* < 0.001). Consistent with the observed growth inhibition, TRI treatment also resulted in a significant reduction in excised tumor weight (Figure 5d,e). The average tumor weight decreased from 0.99 g to 0.53 g in the HepG2 model and from 0.99 g to 0.34 g in the HuH7 model (*p* < 0.01 for both).

Histopathological evaluation of major organs (heart, liver, spleen, lung, and kidney) at the study endpoint (Figure 5f) confirmed TRI’s safety. H&E-stained tissues from treated mice exhibited normal architecture, preserved hepatic lobules, intact myocardium, unaltered renal glomeruli, and typical splenic and pulmonary structure, with no signs of necrosis, inflammation, or degeneration. Two blinded board-certified pathologists, adhering to OECD Guidelines 407, assessed all samples (400× magnification) and detected no drug-related abnormalities. Together with the absence of significant weight loss or overt toxicity (Figure 5b), these findings demonstrate robust anti-tumor efficacy.

## 3. Discussion

Liver cancer remains a significant global public health challenge, contributing substantially to cancer-related morbidity and mortality worldwide. In recent years, the discovery and validation of natural products with potent biological activities have gained considerable attention in medical and biological research [34]. Natural compounds derived from fruits, vegetables, and spices play a significant role in cancer prevention and treatment. These bioactive molecules modulate key mechanisms involved in carcinogenesis, including the suppression of tumor growth, proliferation, inflammation, and oxidative stress [35].

The exploration of bioactive monomers derived from TCM for their anti-tumor properties is an active and rapidly expanding field [14]. Certain compounds exhibit selective cytotoxicity, specifically targeting cancer cells while sparing non-cancerous cells, which may enhance their potential as lead compounds for therapeutic development [36]. This study explored the anti-liver cancer effects of TRI and elucidated its mechanisms of apoptosis induction, providing preclinical insights for further evaluation in liver cancer research.

Dysregulated cell proliferation is a fundamental driver of oncogenesis and tumor progression [37]. In our study, TRI demonstrated dose-dependent cytotoxicity in liver cancer cells (IC_50_ values of 279.9 μg/mL in HepG2 and 444.1 μg/mL in HuH7). To assess cancer cell selectivity, we compared these effects against the THLE-2 cell line, where TRI exhibited lower sensitivity. Beyond cytotoxicity, TRI significantly suppressed colony formation in liver cancer cells, indicating a reduction in their clonogenic potential and long-term proliferative capacity. These findings align with anti-proliferative effects reported for other bioactive herbal compounds [38], and are consistent with our previous observations in breast cancer models [39]. Collectively, these findings demonstrate that TRI can inhibit the proliferative capacity of liver cancer cells while exhibiting limited cytotoxicity toward normal hepatocytes. The consistent response observed across biologically distinct liver cancer cell lines (HepG2 and HuH7) suggests that TRI may exert broad anti-tumor activity, supporting its potential for further mechanistic and translational investigation.

Despite these anticancer effects, the relatively high IC_50_ values highlight potential limitations related to pharmacological potency, achievable systemic exposure, and off-target effects. Therefore, TRI should be interpreted cautiously and is better considered a lead compound requiring further optimization rather than a directly translatable therapeutic agent. Study limitations also include the absence of Ki67 proliferation marker evaluation and transcriptomic profiling (e.g., RNA-seq), which would clarify the molecular pathways modulated by TRI. Future studies should incorporate these analyses using more physiologically relevant liver models to better define TRI’s mechanisms of action and explore its potential to modulate or overcome drug resistance in liver cancer.

Sorafenib, the current first-line systemic therapy for advanced liver cancer, inhibits RAF kinase and angiogenic pathways (e.g., *VEGFR*, *PDGFR*) [40], though its efficacy is often limited by acquired resistance and adverse effects [19,41]. In contrast, TRI appears to exert anti-liver cancer effects through induction of apoptosis and disruption of cell cycle progression [39], as demonstrated in this study. Although a direct experimental comparison with sorafenib was not performed here, reference to reported effects of sorafenib provides useful context. Notably, studies have shown that while sorafenib induces HepG2 cell death via necrosis (2.2%) and apoptosis (17.7% combined early and late), it does not significantly alter cell cycle distribution, consistent with its primary mechanism of *RAF*/*MEK*/*ERK* pathway inhibition rather than cycle arrest [42]. In comparison, agents like crocin and sorafenib/crocin combinations exert anti-proliferative effects through G1 phase arrest [42], highlighting divergent mechanistic strategies. While TRI’s distinct pro-apoptotic and cycle-disruptive effects suggest that TRI may act as a lead compound with distinct or complementary mechanistic effects.

The cell cycle is a fundamental cellular process that regulates cell division and growth. Tumors are often the result of dysregulated cell cycle control, leading to uncontrolled proliferation and the formation of cancerous growths [43]. TRI induced S-phase arrest in liver cancer cells, suggesting that it interacts with pathways controlling cell cycle progression and thereby limits DNA synthesis and proliferation. Other natural compounds, such as Tanshinone and rhoifolin, have been reported to induce cell cycle arrest and caspase-dependent apoptosis in liver cancer cells [34,44]. supporting the relevance of such mechanisms for cancer therapy [45].

**Figure 6:**
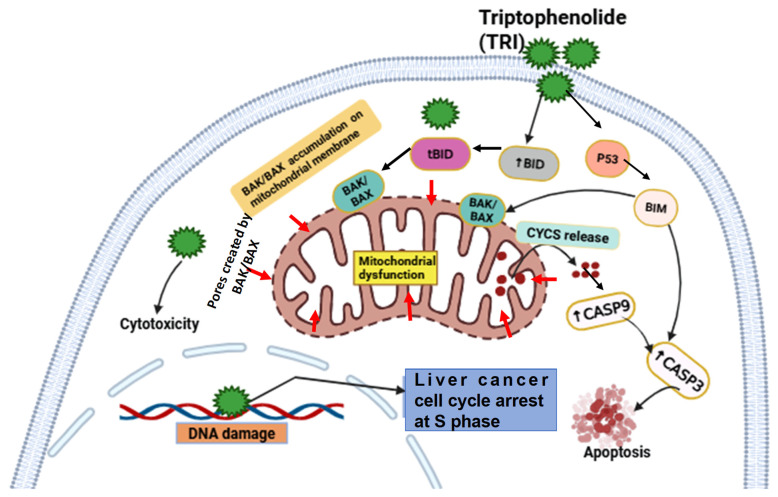
Schematic illustration of the molecular mechanism by which TRI induces apoptosis in hepatocellular carcinoma cells. TRI exerts cytotoxic effects by suppressing liver cancer cell proliferation and inducing S-phase cell cycle arrest. Furthermore, TRI triggers mitochondrial-mediated apoptosis through upregulation of pro-apoptotic genes (including *TP53*, *CASP3*/*9*/*10*, *BAX*, *BAK1*, *BID*, and *BIM*) and corresponding proteins (BAX, BAK1, BID, BIM), leading to mitochondrial membrane permeabilization, cytochrome c release, and subsequent activation of the caspase cascade. These molecular events collectively result in apoptotic cell death, highlighting the multi-level anti-tumor mechanism of TRI in liver cancer.

Apoptosis, a key programmed cell death mechanism, is often dysregulated in cancer, allowing uncontrolled proliferation [46]. In this study, TRI upregulated pro-apoptotic genes, including *CASP3*, *CASP9*, *CASP10*, and *Bcl-2* family members *BAX*, *BAK1*, *BID*, and *BIM*. These proteins facilitate mitochondrial outer membrane permeabilization and promote cytochrome C release, aligning with mechanisms observed for other natural compounds, such as medicarpin [14] (Figure 6). Although our results point to the mitochondrial pathway, direct confirmation via mitochondrial membrane potential assays (e.g., JC-1) was not performed. We acknowledge this limitation and recommend such assays for future mechanistic validation. Further studies are also warranted to determine if TRI modulates upstream regulators such as *p53*, *NF-κB*, or *PI3K*/*AKT*.

In vivo, TRI significantly suppressed tumor growth in subcutaneous liver cancer xenograft models without observable toxicity in major organs, suggesting selective action against tumor cells. However, the molecular mechanisms underlying TRI’s anti-tumor effects were not directly assessed in vivo. Key markers of apoptosis and proliferation, such as Cleaved Caspase-3, Ki67, and γ-H2AX, were not analyzed in tumor tissues, and mitochondrial pathway involvement observed in vitro was not examined in vivo. Additionally, pharmacokinetic parameters and bioavailability of TRI, including plasma concentration, half-life, and tissue distribution, were not assessed, which are important for determining whether the administered concentrations are pharmacologically achievable and for optimizing dosing strategies. Future studies should include comprehensive pharmacokinetic and pharmacodynamic analyses, as well as mechanistic investigations in tumor tissues, to further clarify the translational potential of TRI as a lead compound in liver cancer.

Future investigations should address these limitations by incorporating comprehensive pharmacokinetic and pharmacodynamic analyses, transcriptomic and proteomic profiling, and mechanistic studies in tumor tissues. Such studies will provide a more complete understanding of TRI’s molecular effects and better inform its preclinical translational potential in liver cancer.

## 4. Methods

### 4.1. Cell Lines and Reagents

TRI (HPLC > 98.0%, Desite Biotechnology, Chengdu, China) was dissolved in dimethyl sulfoxide (DMSO; Solarbio, Beijing, China, D8371) and stored at −20 °C. Liver cancer cell lines (HepG2 (CL-0120) and HuH7 (CL-0103)) were obtained from Procell Life Sciences (Wuhan, China) and the THLE-2 (CRL-2706) normal human liver epithelial cell line was obtained from ATCC. Cells were cultured in Dulbecco’s Modified Eagle Medium (DMEM) (HyClone, Logan, UT, USA, SH30022.01) supplemented with 10% fetal bovine serum (FBS; Gibco, Waltham, MA, USA, MT35011CV) and 1% penicillin-streptomycin (PS; Gibco, Waltham, MA, USA, 15140122) at 37 °C in a 5% CO_2_ atmosphere. Upon reaching 90% confluency, cells were detached using 1 mL of trypsin (Biosharp, Hefei, China, BL501A) and subcultured at a 1:3 ratio two to three times per week.

### 4.2. Colony Formation Assay

A clonogenic survival assay was performed. HepG2 and HuH7 cells were seeded in 6-well plates (1000 cells/well) and allowed to adhere for 24 h. The cells were then treated with 200 μM TRI or vehicle control for 24 h. After treatment, the medium was replaced with fresh, drug-free medium, and the cells were cultured for approximately 14 days to allow colony formation. The resulting colonies were washed with ice-cold PBS, fixed with 70% ethanol, and then stained with 1% crystal violet solution (Merck, Darmstadt, Germany, C.I. 42555) for 20 min. Following staining, the plates were gently washed with tap water to remove excess dye. The colonies were air-dried, imaged, and quantified using ImageJ software (2.1.0: NIH, Bethesda, MD, USA).

### 4.3. Cell Proliferation Assay

HepG2, HuH7, and THLE-2 cells were seeded into 96-well plates at 5 × 10^3^ cells/well (100 μL DMEM medium) and allowed to adhere for 4–6 h. Following attachment, cells were treated with varying concentrations of TRI (50–500 µg/mL) or DMSO (control) for 24 or 48 h. At each time point, the medium was replaced with 100 μL fresh DMEM medium containing 10% CCK-8 reagent (Dojindo, Kumamoto, Japan), and plates were incubated for 1.5–2 h at 37 °C. Absorbance at 450 nm was measured using a Multiskan FC microplate reader (Thermo Fisher Scientific, Waltham, MA, USA). Cell viability was calculated as:

Cell viability (%) = [(Aexperimental − Ablank)/(Acontrol − Ablank)] × 100

The half-maximal inhibitory concentration (IC_50_) was calculated using nonlinear regression analysis in GraphPad Prism 8.2.0.

### 4.4. Cell Cycle Analysis

HepG2 and HuH7 cells (2 × 10^5^ cells/well) were seeded in 6-well plates, synchronized in serum-free medium for 24 h, and treated with TRI (200 or 400 µg/mL) for 48 h. Cells were fixed in 70% ethanol at −20 °C for 24 h, washed with PBS, and stained with 50 µg/mL RNase A and 25 µL propidium iodide (PI; 1 mg/mL; Beyotime Biotech, Shanghai, China). Cell cycle distribution was analyzed using a BD Biosciences flow cytometer (561 nm excitation) and FlowJo 10.4 software.

### 4.5. Apoptosis Assay

To evaluate apoptosis, cells were plated at a density of 2 × 10^5^ cells/well in 6-well plates. Following a 48 h treatment with 150 μg/mL TRI or a 0.1% DMSO vehicle control, cells were harvested, washed with cold PBS, and resuspended in 195 μL of binding buffer. The cell suspensions were subsequently stained with 5 μL of Annexin V-FITC and 10 μL of propidium iodide (PI, 20 μg/mL) for 20 min at room temperature in the dark. Samples were analyzed on a BD FACSVerse flow cytometer, with a minimum of 10,000 events recorded per sample. Fluorescence was detected using a 488 nm laser, with FITC emission collected at 530/30 nm and PI at 585/40 nm. Data were processed with FlowJo V10 software to determine the percentages of viable (Annexin V^−^/PI^−^), early apoptotic (Annexin V^+^/PI^−^), and late apoptotic/necrotic (Annexin V^+^/PI^+^) cell populations. The Annexin V-FITC apoptosis detection kit was procured from Beyotime Biotechnology.

### 4.6. Quantitative Real-Time PCR

To analyze gene expression, HepG2 and HuH7 cells (2 × 10^5^ cells/well) were treated for 48 h with either TRI (at IC_50_ doses of 200 μg/mL) or a 0.1% DMSO vehicle control. We isolated total RNA with the RNAprep pure Cell Kit and reverse-transcribed it into cDNA using the FastKing RT Kit (both from Tiangen, Beijing, China), following the supplied protocols. Quantitative PCR was performed in technical triplicates on a QuantStudio1 system (Thermo Fisher Scientific, Waltham, MA, USA) with PowerUp SYBR Green Master Mix. The GAPDH gene served as an internal control for normalizing expression levels, which were calculated via the ΔΔCt method. Results are representative of three independent biological experiments. The sequences for all primers used are listed in Table 1.

### 4.7. Western Blot Analysis

HepG2 and HuH7 cells (2 × 10^5^ cells/well) were treated with 200 µg/mL TRI for 48 h. Proteins were extracted, separated by SDS-PAGE, and transferred to PVDF membranes (0.45 μm; Millipore, Bedford, MA, USA). Membranes were blocked with 7% skim milk 1 h at room temperature and then incubated overnight at 4 °C with primary antibodies from Cell Signaling Technology (Danvers, MA, USA) (1:1000 dilution in 5% BSA/TBST): rabbit monoclonal antibodies against BID, BIM, BAX, BAK1, and GAPDH (loading control). Following three 10 min TBST washes, membranes were incubated with HRP-conjugated secondary antibodies (goat anti-rabbit IgG or goat anti-mouse IgG, 1:5000; Nakasugi Golden Bridge, Beijing, China) for 1 h at room temperature. Proteins were visualized using an ECL kit (PE0010, Solarbio, Beijing, China) and quantified using ImageJ 2.1.0.

### 4.8. Liver Cancer Cell Subcutaneous Transplantation

BALB/c nude mice (*n* = 20, female, 6 weeks old, 15 ± 2 g; Spectrum, Beijing, China) were subcutaneously injected with HepG2 or HuH7 cells (5 × 10^6^ cells) in the right shoulder. When tumors reached 100 mm^3^, mice were randomized into control (1% DMSO, 100 µL, *n* = 5) and TRI-treated (10 mg/kg, 100 µL, *n* = 5) groups. The selected in vivo dose of 10 mg/kg was based on previously reported effective dose ranges for structurally related natural diterpenoids and was further supported by the observed antitumor efficacy and absence of significant toxicity in our xenograft models. This dose is consistent with commonly used ranges for preclinical evaluation of natural compounds [34,47,48]. TRI was administered intraperitoneally every three days. Tumor volumes were measured every 3 days using digital calipers and calculated using the formula: (length × width^2^)/2. Mice were humanely euthanized when tumor volumes reached 1000 mm^3^ or if signs of distress were observed, in accordance with institutional guidelines. Excised tumors were weighed, photographed, and processed for subsequent analyses. Survival rates were monitored and analyzed using Kaplan–Meier curves (GraphPad Prism 8.2.0). TRI was prepared as a 50 mM stock solution in DMSO, stored at −20 °C, and freshly diluted for each treatment.

### 4.9. Histopathological Analysis

Major organs from experimental and control mice were collected, fixed in formalin, paraffin-embedded, and sectioned at appropriate thickness. Tissue sections were first deparaffinized using xylene and rehydrated through a graded ethanol series (100%, 95%, 85%, and 75%) followed by rinsing in distilled water. The sections were then stained with hematoxylin solution (Abcam, Cambridge, UK, ab245880) to visualize nuclei and rinsed in running tap water. Differentiation and bluing steps were performed according to the manufacturer’s instructions to enhance nuclear contrast. Subsequently, the sections were counterstained with eosin to visualize cytoplasmic and extracellular structures. After staining, the sections were dehydrated through graded ethanol, cleared in xylene, and mounted with a coverslip using mounting medium. Histopathological evaluation was performed under a light microscope to assess tissue morphology and detect any treatment-related abnormalities.

### 4.10. Statistical Analysis

All experiments included a minimum of three independent biological replicates. Data are expressed as mean ± standard deviation (SD). Statistical analyses were performed using GraphPad Prism 8.2.0 (GraphPad Software, San Diego, CA, USA). Specifically, GraphPad Prism was used to analyze all quantitative data, including cell proliferation assays and in vivo mouse model studies. Differences between two groups were assessed by an unpaired Student’s *t*-test. For comparisons across multiple groups, one-way or two-way ANOVA was employed, followed by appropriate post hoc tests. Data from flow cytometry experiments (cell cycle and apoptosis) were analyzed using FlowJo software (Version 10.8.1, BD Life Sciences, Franklin Lakes, NJ, USA). Western blot band intensities and liver cancer colonies were quantified using ImageJ 1.8.0 (NIH, Bethesda, MD, USA). A *p*-value of less than 0.05 was considered statistically significant, denoted as follows: * *p* < 0.05, ** *p* < 0.01, *** *p* < 0.001, and **** *p* < 0.0001

## 5. Conclusions

Collectively, this study demonstrates that TRI exerts potent anti-proliferative and pro-apoptotic effects on liver cancer cells in vitro and suppresses tumor growth in vivo with minimal observed toxicity. These effects are mediated, at least in part, through mitochondrial-dependent apoptotic pathways and cell cycle arrest at the S phase. While these findings provide important preclinical insights, further studies are required to characterize TRI’s pharmacokinetics, molecular targets, and long-term safety, and to evaluate its efficacy in combination with established liver cancer therapies. Such investigations will help clarify its potential for future translational development.

## Figures and Tables

**Table 1:** Primer list of genes used for qRT-PCR.

Gene	Forward Primer Sequence (5′-3′)	Reverse Primer Sequence (5′-3′)
*TP53*	TGGAGTCCGAGGAGAAGTTG	GCTGAGGAGAAGCTGAGCAA
*GAPDH*	AAGGTGAAGGTCGGAGTCAA	GGAAGATGGTGATGGGATTT
*CASP9*	CGAACTAACAGGCAAGCA	AATCCTCCAGAACCAATGTC
*CASP3*	AAGCGAATCAATGGACTCT	TGTACCAGACCGAGATGT
*CASP10*	TAGGATTGGTCCCCAACAAGA	GAGAAACCCTTTGTCGGGTGG
*BIM*	CTGAGTGTGACCGAGAAG	GATTACCTTGTGGCTCTGT
*BID*	ATGGACCGTAGCATCCCTCC	GTAGGTGCGTAGGTTCTGGT
*BAX*	CCTTTTGCTTCAGGGTTTCA	CAGTTGAAGTTGCCGTCAGA
*BAK1*	TCTGGCCCTACACGTCTACC	ACAAACTGGCCCAACAGAAC

## Data Availability

The data that support the findings of this study are available from the corresponding author by E-mail upon reasonable request.
